# Gastric Spindle Cell Neoplasm With LMNA::NTRK1 Fusion: A Rare Mimic of Wild‐Type GIST

**DOI:** 10.1155/cris/5492086

**Published:** 2026-10-21

**Authors:** Yoonji Seo, Christopher Leung, Mitali Fadia, Christopher Seng Hong Lim, Sivakumar Gananadha

**Affiliations:** ^1^ School of Medicine and Psychology The Australian National University Canberra 2601 Australian Capital Territory Australia anu.edu.au; ^2^ Department of Surgery The Canberra Hospital Yamba Drive Garran 2605 Australian Capital Territory Australia health.act.gov.au; ^3^ Department of Anatomical Pathology The Canberra Hospital Yamba Drive Garran 2605 Australian Capital Territory Australia health.act.gov.au

## Abstract

Neurotrophic tyrosine receptor kinase‐rearranged spindle cell neoplasms (NTRK‐SCNs) are rare mesenchymal tumours defined by oncogenic rearrangements involving NTRK genes. NTRK fusions are exceedingly rare in the gastrointestinal tract and can closely resemble wild‐type gastrointestinal stromal tumours (GISTs), posing a diagnostic challenge in the absence of molecular testing. We report a case of a 36‐year‐old woman with a distal gastric mass initially suspected to be a GIST. Surgical resection revealed a spindle cell tumour with low mitotic activity and perivascular hyalinisation. Immunohistochemistry demonstrated expression of CD34, S100 and pan‐TRK and was negative for KIT/CD117 and DOG1. Molecular testing confirmed an LMNA::NTRK1 fusion, consistent with NTRK‐SCN. This case highlights the importance of considering NTRK‐SCN in KIT/DOG1‐negative gastric spindle cell tumours.

## 1. Introduction

Neurotrophic tyrosine receptor kinase‐rearranged spindle cell neoplasms (NTRK‐SCNs) are a recently recognised subset of mesenchymal tumours characterised by oncogenic NTRK gene fusions [1]. The NTRK1, NTRK2 and NTRK3 genes encode tropomyosin receptor kinases (TRK) TrkA, TrkB and TrkC, respectively [2]. Aberrant fusions involving these genes lead to constitutive activation of TRK signalling, promoting oncogenesis [3]. While they are considered defining molecular features in certain tumours such as infantile fibrosarcoma [4], NTRK fusions tend to arise from the soft tissues of the extremities or trunk. Their occurrence in visceral organs is uncommon, especially in the gastrointestinal tract and exceptionally rare in the stomach.

The diagnostic approach to a gastric mass involves contrast‐enhanced computed tomography (CT), followed by endoscopy, biopsy and histopathological analysis. Although pan‐TRK immunohistochemistry can screen for NTRK gene rearrangements, molecular profiling is necessary to confirm the presence and identity of specific fusions. Risk stratification and management are guided by tumour size, location and mitotic count. While complete surgical resection remains the primary treatment for low‐grade, localised disease, systemic therapies such as TRK inhibitors are available for the treatment of unresectable or metastatic tumours [4].

A key diagnostic challenge is distinguishing gastric NTRK‐SCN from gastrointestinal stromal tumours (GISTs), especially wild‐type variants that lack KIT or PDGFRA mutations. While both entities can histologically appear as spindle cell neoplasms, NTRK‐SCNs lack KIT and DOG1 expression and demonstrate pan‐TRK positivity, often with co‐expression of CD34 and S100 [5]. In contrast, most GISTs exhibit KIT or DOG1 on immunohistochemistry. Wild‐type GISTs lacking KIT or DOG1 expression may harbour alternative pathway mutations but rarely NTRK fusions. The malignant potential of gastrointestinal NTRK‐SCNs ranges from low‐grade tumours with an indolent course to aggressive tumours with metastatic features, although the latter is rarer [6].

NTRK‐SCNs are emerging entities with fewer than five cases reported in the stomach. Therefore, we are yet to understand their clinical behaviour, prognosis and treatment. This case highlights the utility of molecular diagnostics in identifying a gastric NTRK‐SCN initially suspected to be a wild‐type GIST.

## 2. Case Presentation

A woman in her mid‐30 s presented with a 6‐year history of upper abdominal pain with no associated gastrointestinal or systemic symptoms. Prior surgical history included hysterectomy. The physical examination was unremarkable, with no palpable abdominal mass. Initial abdominal ultrasonography revealed a distal gastric mass, which was further investigated with contrast‐enhanced CT of the chest, abdomen and pelvis (Figure 1) and endoscopy (Figure 2). CT of the abdomen revealed a well‐circumscribed, enhancing lesion at the pylorus measuring 36 mm × 32 mm × 39 mm. Endoscopic biopsy revealed a low‐grade spindle cell tumour. The patient elected to undergo surgical resection due to marginal tumour growth on surveillance imaging.

**Figure 1:**
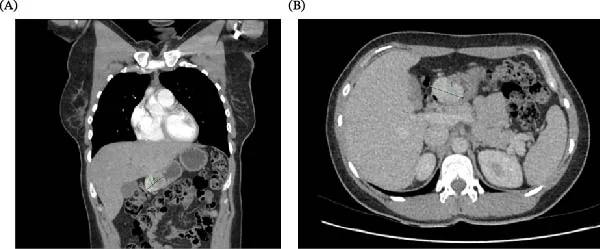
Coronal (A) and axial (B) intravenous contrast‐enhanced CT scan exhibiting a well‐circumscribed enhancing lesion at the pylorus measuring 36 mm × 32 mm × 39 mm.

**Figure 2:**
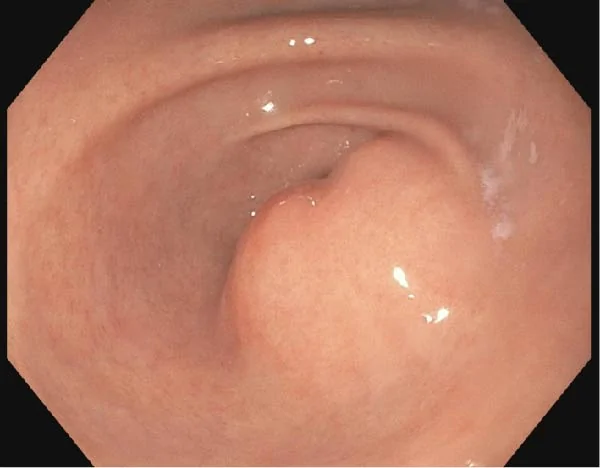
Endoscopic image of the pyloric mass.

A laparoscopic distal gastrectomy with Roux‐en‐Y reconstruction was performed. Macroscopic examination of the resected specimen revealed a round, well‐demarcated solid mass measuring 45 mm × 32 mm × 25 mm with a central cystic cavity measuring 23 mm × 7 mm × 36 mm. There were no areas of haemorrhage or necrosis. Microscopic evaluation (Figure 3) revealed a relatively uniform spindle cell population arranged haphazardly within a collagenous stroma, with prominent interstitial fibrosis and perivascular ring‐like hyalinisation. The tumour infiltrated the submucosa and muscularis propria with chronic inflammatory infiltrate. The mitotic count was low (2 per 50 high‐power fields), correlating with a low proliferative index (Ki‐67 index of 1%). Resection margins were negative, and no associated lymph nodes were identified.

**Figure 3:**
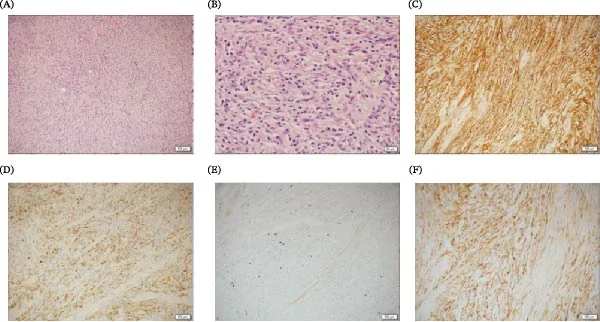
Histological and immunohistochemical features of the gastric LMNA::NTRK1‐rearranged spindle cell neoplasm, with scale bars in each panel. (A, B) Haematoxylin and eosin‐stained sections at low and high magnification, showing bland spindle cells within a collagenous stroma with perivascular hyalinisation. (C) Diffuse CD34 expression. (D) Patchy S100 expression. (E) Absence of CD117 expression in tumour cells, with positively stained mast cells. (F) Positive pan‐TRK immunostaining.

Immunohistochemistry demonstrated diffuse CD34 expression, focal weak S100 staining and pan‐TRK expression. The tumour was negative for KIT/CD117, DOG1, SMA, desmin, AE1/AE3, SOX10, STAT6 and CD31. Molecular profiling was performed to further characterise the lesion and distinguish GIST and other gastric spindle cell neoplasms. This was conducted using the OncoStrands Essential Panel with the Oncomine Precision Assay (Thermo Fisher Scientific) using the Genexus Integrated Sequencer platform (Lifestrands Genomic). This assay assessed DNA hotspot alterations, copy‐number alterations and RNA fusions across a targeted panel of genes associated with malignancies. No pathogenic mutations were detected in the KIT, PDGFRA and BRAF genes, and no fusion transcripts were detected in FGFR1/2/3, NTRK2 or NTRK3. However, the assay identified an LMNA::NTRK1 fusion, confirming the diagnosis of an NTRK‐rearranged spindle cell neoplasm.

The patient recovered uneventfully and was discharged 4 days after surgery. She was followed up 1 month postoperatively with no clinical concerns. The multidisciplinary team recommended 12‐month follow‐up with CT imaging of the chest, abdomen and pelvis. This recommendation was based on complete resection with negative margins, low mitotic activity and Ki‐67, and no evidence of metastatic disease. Given their rarity, the optimal frequency and duration of postoperative surveillance for gastrointestinal NTRK‐SCNs have not been established. Therefore, this was an individual multidisciplinary decision rather than a validated surveillance plan. The patient remained clinically well on follow‐up with no evidence of local recurrence or metastatic disease on CT imaging. In the event of recurrence, further surgery would be considered if the disease remained resectable. Advanced or unresectable disease could be considered for TRK‐directed systemic therapy.

## 3. Discussion

NTRK‐SCNs are defined by oncogenic NTRK fusion mutations with characteristic histological features, including pan‐TRK positivity, coexpression of S100 and CD34, and absence of KIT, DOG1 and SOX10 [7]. Microscopically, they often exhibit spindle cell morphology with perivascular hyalinisation. Among adults, the most common fusions include ETV6::NTRK3 (26.4%), followed by TPM3::NTRK1 (21.5%) and LMNA::NTRK1 (9.5%). NTRK1 fusions are most common within the gastrointestinal tract [7]. LMNA::NTRK1 fusion tumours have been reported in the stomach, small intestine and colon (Table 1). These tumours demonstrate variable biological behaviour; some are indolent and low‐grade, while others display metastatic potential [6].

**Table 1:** Gastrointestinal LMNA::NTRK1 fusion tumours reported in literature.

Reference	Tumour type	Location	Age, sex	Outcome
Suurmeijer et al. [8]	Spindle cell neoplasm	Stomach	18, M	Treated with larotrectinib but died of disease (25 months)
Atiq et al. [6]	Spindle cell neoplasm	Stomach	4, F	No evidence of disease (3.5 months)
Rahim et al. [9]	Spindle cell neoplasm	Small intestine	Early 20 s, F	No evidence of disease (3 months)
Gao et al. [7]	Spindle cell neoplasm	Colon	6, F	No evidence of disease
		Small intestine	63, M	No evidence of disease
		Stomach	48, F	No evidence of disease

Our case demonstrates a low‐grade LMNA::NTRK1‐rearranged SCN. While initially considered a wild‐type GIST, the immunophenotype and microscopic morphology were found to be consistent with NTRK‐SCN. Although rare NTRK fusions have been detected in GIST‐like tumours, it is unclear whether these represent true GISTs or misclassified NTRK‐SCNs [10]. In wild‐type GISTs, alternative mutations such as succinate dehydrogenase deficiency or RAS‐pathway mutations are more frequently observed than NTRK fusions [11]. The absence of KIT or DOG1 expression alongside CD34, S100 and pan‐TRK positivity should raise suspicion for NTRK‐SCN [6]. Hence, NTRK‐SCN should be considered in KIT/PDGFRA‐negative gastrointestinal mesenchymal tumours with an atypical morphology or immunophenotype.

The differential diagnosis for gastric spindle cell lesions is broad and includes other mesenchymal entities. Gastric schwannomas can demonstrate S100 positivity but typically exhibit diffuse SOX10 expression and lack the CD34 and pan‐TRK positivity observed in this case [12]. Solitary fibrous tumours, which can present as well‐circumscribed gastric masses, were considered less likely by the absence of STAT6 expression and characteristic patternless architecture [13]. Although inflammatory fibroid polyps may express CD34, the overall morphological and molecular features, including the lack of PDGFRA mutations, were favourable for an NTRK‐rearranged spindle cell neoplasm [14]. Leiomyoma and leiomyosarcoma were less likely diagnoses due to the lack of smooth muscle morphology and the absence of SMA and desmin expression [15]. Plexiform fibromyxomas exhibit spindle cell morphology and occur most commonly in the antrum and pylorus; however, the tumour lacked plexiform growth in a myxoid stroma and was SMA‐negative, whereas CD34 and S100 were expressed [16]. Desmoid‐type fibromatosis was also considered less likely due to CD34 and S100 expression, SMA negativity and the demonstrated fusion [17].

LMNA::NTRK1 fusion tumours have previously been reported in the stomach, small intestine and colon (Table 1). Suurmeijer et al. [8] described a gastric spindle cell neoplasm in an 18‐year‐old man that followed an aggressive course, whereas Atiq et al. [6] reported a gastric tumour in a 4‐year‐old girl without disease recurrence at 3.5 months post‐resection. Rahim et al. [9] reported an intestinal spindle cell neoplasm in a woman in her early 20 s with no evidence of disease after 3 months. Gao et al. [7] reported three cases of LMNA::NTRK1 fusion tumours in patients of varying ages, all without disease recurrence. Hence, the literature suggests variable biological behaviour and occurrence of this rare tumour across variable age groups.

The literature also suggests that gastrointestinal NTRK fusion tumours exhibit significant morphological diversity. Atiq et al. [6] described a morphological spectrum ranging from infantile fibrosarcoma‐like tumours to low‐grade CD34/S100‐positive spindle cell neoplasms, as well as unclassified high‐grade spindle cell sarcomas. Gao et al. [7] similarly identified both low‐grade tumours exhibiting haphazard morphology, often with stromal collagen and ring‐like perivascular hyalinisation, as well as high‐grade sarcomas with atypical cytology, increased mitotic activity and increased cellularity. The morphology of the tumour discussed in this case is most consistent with a low‐grade entity, but the small number of reported cases and limited follow‐up data preclude a reliable conclusion regarding prognosis.

Surgical resection remains the first‐line treatment for localised gastrointestinal tumours, including NTRK‐SCN. In the absence of metastases or unresectable disease, complete excision may be performed with curative intent, particularly for low‐grade tumours. However, the clinical behaviour of NTRK‐SCN appears heterogeneous, with some tumours exhibiting high‐grade features or recurring following initial surgical management. In such cases, systemic therapies may be warranted. Selective TRK inhibitors such as larotrectinib and entrectinib have been shown to produce high response rates across various types of NTRK‐fusion‐positive neoplasms, including colon cancer and GISTs [18, 19]. The National Comprehensive Cancer Network (NCCN) guidelines recommend TRK inhibitor therapy for advanced NTRK‐rearranged solid tumours [20]. Molecular profiling is therefore significant not only for diagnosis but also for making treatment decisions, particularly when considering systemic therapy in advanced or recurrent disease. This case demonstrates the importance of considering NTRK‐SCN in KIT/DOG1‐negative gastric tumours. Integration of morphology, immunohistochemistry and molecular testing is essential for accurate diagnosis. Recognition of an NTRK fusion also has potential therapeutic relevance in unresectable, recurrent or metastatic disease.

## Funding

The authors have nothing to report. Open access publishing facilitated by Australian National University, as part of the Wiley ‐ Australian National University agreement via the Council of Australasian University Librarians.

## Consent

Written informed consent was obtained from the patient for the publication of this case report and any accompanying images.

## Conflicts of Interest

The authors declare no conflicts of interest.

## Data Availability

The data that support the findings of this study are available upon request from the corresponding author. The data are not publicly available due to privacy or ethical restrictions.

## References

[1] Board WCoTE , Soft Tissue and Bone Tumours, 2020, 5th edition, International Agency for Research on Cancer (IARC).

[2] Nakagawara A. , Trk Receptor Tyrosine Kinases: A Bridge Between Cancer and Neural Development, Cancer Letters. (2001) 169, no. 2, 107–114, 10.1016/S0304-3835(01)00530-4. 11431098

[3] Amatu A. , Sartore-Bianchi A. , and Siena S. , NTRK Gene Fusions as Novel Targets of Cancer Therapy Across Multiple Tumour Types, ESMO Open. (2016) 1, no. 2, 10.1136/esmoopen-2015-000023. PMC5070277 27843590

[4] Cocco E. , Scaltriti M. , and Drilon A. , NTRK Fusion-Positive Cancers and TRK Inhibitor Therapy, Nature Reviews Clinical Oncology. (2018) 15, no. 12, 731–747, 10.1038/s41571-018-0113-0. PMC6419506 30333516

[5] Gatalica Z. , Xiu J. , Swensen J. , and Vranic S. , Molecular Characterization of Cancers With NTRK Gene Fusions, Modern Pathology. (2019) 32, no. 1, 147–153, 10.1038/s41379-018-0118-3. 30171197

[6] Atiq M. A. , Davis J. L. , and Hornick J. L. , et al.Mesenchymal Tumors of the Gastrointestinal Tract With NTRK Rearrangements: A Clinicopathological, Immunophenotypic, and Molecular Study of Eight Cases, Emphasizing Their Distinction From Gastrointestinal Stromal Tumor (GIST), Modern Pathology. (2021) 34, no. 1, 95–103, 10.1038/s41379-020-0623-z. 32669612

[7] Gao X. , Xu S. , Zhu P. , Lao I. W. , Yu L. , and Wang J. , Primary NTRK-Rearranged Spindle Cell Neoplasm of the Gastrointestinal Tract: A Clinicopathological and Molecular Analysis of 8 Cases, American Journal of Surgical Pathology. (2024) 48, no. 5, 623–631, 10.1097/PAS.0000000000002202. 38525823

[8] Suurmeijer A. J. H. , Dickson B. C. , and Swanson D. , et al.A Novel Group of Spindle Cell Tumors Defined by S100 and CD34 Co-Expression Shows Recurrent Fusions Involving RAF1, BRAF, and NTRK1/2 Genes, Genes, Chromosomes and Cancer. (2018) 57, no. 12, 611–621, 10.1002/gcc.22671. PMC6746236 30276917

[9] Rahim S. , Alkhaldi S. S. , Alasousi K. , and Ali R. H. , Intestinal *LMNA*: *NTRK1*-Fused Spindle Cell Neoplasm With S100 and CD34 Coexpression: A New Case, BMJ Case Reports. (2022) 15, no. 11, 10.1136/bcr-2022-251270. PMC9660640 36357104

[10] Lee J. H. , Shin S.-J. , and Choe E.-A. , et al.Tropomyosin-Related Kinase Fusions in Gastrointestinal Stromal Tumors, Cancers. (2022) 14, no. 11, 10.3390/cancers14112659, 2659. PMC9179593 35681640

[11] IJzerman N. S. , Drabbe C. , and den Hollander D. , et al.Gastrointestinal Stromal Tumours (GIST) in Young Adult (18–40 Years) Patients: A Report From the Dutch GIST Registry, Cancers. (2020) 12, no. 3, 10.3390/cancers12030730, 730. PMC7140070 32244864

[12] Voltaggio L. , Murray R. , Lasota J. , and Miettinen M. , Gastric Schwannoma: A Clinicopathologic Study of 51 Cases and Critical Review of the Literature, Human Pathology. (2012) 43, no. 5, 650–659, 10.1016/j.humpath.2011.07.006. PMC3305846 22137423

[13] Doyle L. A. , Vivero M. , Fletcher C. D. M. , Mertens F. , and Hornick J. L. , Nuclear Expression of STAT6 Distinguishes Solitary Fibrous Tumor From Histologic Mimics, Modern Pathology. (2014) 27, no. 3, 390–395, 10.1038/modpathol.2013.164. 24030747

[14] Liu T.-C. , Lin M.-T. , Montgomery E. A. , and Singhi A. D. , Inflammatory Fibroid Polyps of the Gastrointestinal Tract: Spectrum of Clinical, Morphologic, and Immunohistochemistry Features, American Journal of Surgical Pathology. (2013) 37, no. 4, 586–592, 10.1097/PAS.0b013e31827ae11e. 23426127

[15] Alpert L. , Al-Sabti R. , and Graham R. P. , et al.Smooth Muscle Tumors of the Gastrointestinal Tract: An Analysis of Prognostic Features in 407 Cases, Modern Pathology. (2020) 33, no. 7, 1410–1419, 10.1038/s41379-020-0492-5. PMC8405135 32051556

[16] Miettinen M. , Makhlouf H. R. , Sobin L. H. , and Lasota J. , Plexiform Fibromyxoma: A Distinctive Benign Gastric Antral Neoplasm Not to Be Confused With a Myxoid GIST, American Journal of Surgical Pathology. (2009) 33, no. 11, 1624–1632, 10.1097/PAS.0b013e3181ae666a. 19675452

[17] Lee J. C. , Curtis D. , Williamson J. B. , and Ligato S. , Gastric Desmoid Fibromatosis – Report of a Rare Mimic of Gastrointestinal Stromal Tumor, Cureus. (2021) 13, no. 11, 10.7759/cureus.19614. PMC8673680 34926078

[18] Doebele R. C. , Drilon A. , and Paz-Ares L. , et al.Entrectinib in Patients With Advanced or Metastatic NTRK Fusion-Positive Solid Tumours: Integrated Analysis of Three Phase 1–2 Trials, The Lancet Oncology. (2020) 21, no. 2, 271–282, 10.1016/S1470-2045(19)30691-6. PMC7461630 31838007

[19] Drilon A. , Laetsch T. W. , and Kummar S. , et al.Efficacy of Larotrectinib in *TRK* Fusion–Positive Cancers in Adults and Children, New England Journal of Medicine. (2018) 378, no. 8, 731–739, 10.1056/NEJMoa1714448. PMC5857389 29466156

[20] Network NCC , Soft Tissue Sarcoma (Version 1.2025), 2025, https://www.nccn.org/guidelines/guidelines-detail?category=1&id=1464.

